# Derivation and Validation of the Management and Supervision Tool (MaST) Risk of Crisis (RoC) Algorithm Using Electronic Health Record (EHR) Data

**DOI:** 10.1192/bjo.2022.229

**Published:** 2022-06-20

**Authors:** Rashmi Patel, Jamie Oram, Nick Hebden, Zo Payne, Martin Morse, Caroline Gadd

**Affiliations:** 1 Holmusk, London, United Kingdom; 2 Institute of Psychiatry, Psychology & Neuroscience, King's College London, London, United Kingdom

## Abstract

**Aims:**

The Management and Supervision Tool (MaST) helps NHS mental health care professionals identify patients who are most likely to need psychiatric hospital admission or home treatment, due to severe mental illness, through a Risk of Crisis (RoC) algorithm driven by electronic health record (EHR) data analytics. We describe the derivation and validation of the MaST RoC algorithm, and its implementation to support preventative mental healthcare in the NHS.

**Methods:**

The RoC algorithm was developed and evaluated with EHR data from six UK NHS trusts using Ordered Predictor List propensity scores informed by a priori weightings from pre-existing literature, as well as real-world evidence evaluating the associations of clinical risk factors with mental health crisis using NHS EHR data. Mental health crisis was defined as admission to a psychiatric hospital or acceptance to a community crisis service within a 28-day period. Predictor variables included age, gender, accommodation status, employment status, Mental Health Act (MHA) status (under section or Community Treatment Order), and previous mental health service contacts (including hospital admissions and crisis services). Data were analysed using Ordered Predictor List propensity scores. The algorithm was derived using structured EHR data from 2,620 patients in a single NHS trust and externally validated using data from 107,879 patients in five other NHS trusts. Qualitative and quantitative data on feasibility, acceptability and system efficiency impacts of MaST implementation were obtained through staff surveys and local audits.

**Results:**

The factors associated with greatest propensity for mental health crisis included recent previous crisis, multiple previous crises, higher number of mental health service contacts in recent weeks, MHA section, accommodation status and employment status. The RoC algorithm identified 64% and 80% crises in its top quintile. Sentiment analysis of staff surveys suggested that the use of MaST improved productivity by reducing time taken to access patient information to support caseload management that was previously difficult to obtain through manual review of EHRs. The systems efficiency audit revealed a reduction in duration of crisis and inpatient admissions following MaST implementation.

**Conclusion:**

The MaST RoC algorithm supports the identification of people more likely to use crisis services in NHS mental health trusts, is feasible to implement, and improves systems efficiency. EHR-derived algorithms can support real-world clinical practice to improve outcomes in people receiving NHS mental healthcare.

